# Potassium-solubilizing bacteria from tropical forest soils enhance potassium and phosphorus uptake in maize

**DOI:** 10.3389/fmicb.2026.1788341

**Published:** 2026-05-14

**Authors:** Pragya Saxena, Mandira Barman, Shaloo Verma, Sudipta Das, Jagriti Yadav, Murugan Kumar, Arjun Singh, Surender Singh, Hillol Chakdar, Anil Kumar Saxena, Alok Kumar Srivastava

**Affiliations:** 1 ICAR-National Bureau of Agriculturally Important Microorganisms (ICAR-NBAIM), Mau, India; 2 Department of Soil Science and Agricultural Chemistry, ICAR-Indian Agricultural Research Institute (ICAR-IARI), New Delhi, India; 3 ICAR-Central Soil Salinity Research Institute (CSSRI), Regional Station, Lucknow, Uttar Pradesh, India; 4 Department of Microbiology, Central University of Haryana, Mahendragarh, Haryana, India

**Keywords:** *Bacillus tequilensis*, maize, *Priestia aryabhattai*, potassium solubilizing bacteria, *Priestia megaterium*

## Abstract

**Introduction:**

The continued use of chemical fertilizers to enhance productivity often disturbs the ecological balance of the soil. Integrated nutrient management involving beneficial microorganisms is a promising option for the sustainable maintenance of soil health. In the present study, we aimed to identify potassium (K)-solubilizing bacteria from the tropical evergreen forests of Meghalaya, India, and evaluate their growth-enhancing potential in maize under controlled conditions.

**Methods:**

When tested for K and P solubilizing activity *in vitro*, *Bacillus tequilensis* NPH2 showed maximum K solubilization (4.71 ± 0.17 mg L^−1^), while *Priestia aryabhattai* NPH4 showed the highest rock phosphate (280 ± 5.2 mg L^−1^) solubilization. All the strains were found to produce varying amounts of indole-3-acetic acid, while only two strains (NPH1 and NPH2) produced siderophores. The effect of seed inoculation on plant biometric parameters of maize was variable.

**Results:**

K and phosphorus (P) contents were significantly improved by all the strains. During the pot experiment, treatment with *P. aryabhattai* NPH4 resulted in the highest available K (35 ± 1.0 g kg^−1^ dry weight) and P (3.13 ± 0.01 g kg^−1^ dry weight) in maize. Shoot growth of *P. aryabhattai* NPH4-inoculated maize plants was significantly better than that of uninoculated plants and the positive control.

**Discussion:**

*P. aryabhattai* NPH4 was able to survive under acidic to neutral pH, up to 4% NaCl, and temperatures from 15 °C to 45 °C, which can help it to adapt to a wide range of edapho-climatic conditions. The results suggest that *P. aryabhattai* NPH4 has significant potential as a K-solubilizing microorganism with multifarious plant growth-promoting traits.

## 1 Introduction

Potassium (K) is a key macronutrient that plays a remarkable role in plant growth, metabolism, and development. This macronutrient is responsible for adjusting cellular osmotic pressure, activating enzymes, utilizing nitrogen, and synthesizing proteins and sugars (Zhang and Kong, 2014). However, various studies have reported that rhizobacteria play a crucial role in enhancing nutrient uptake from the soil, which directly contributes to plant growth and development (Rani et al., 2022, 2023). Although soil potassium levels were once considered sufficient, approximately 20.0–50.0% of arable land is now found to have low to medium potassium content (Brownlie et al., 2024). Generally, K in soil is present in the following forms: (i) dissolved K, (ii) exchangeable K bound to negatively charged colloidal soil particles, (iii) non-exchangeable K trapped between mineral layers, and (iv) mineral K present in primary minerals, such as mica and feldspar (Annapurna and Kadam, 2016). Dissolved and exchangeable K are easily available for plant uptake but contribute only <2.0% of total soil K (Etesami et al., 2017). More than 90.0% of soil potassium is present as mineral K, which is an unavailable form of potassium, creating a paradox in soils that are K-rich and at the same time K-deficient (Gurav et al., 2019). Making the unavailable potassium in minerals accessible to plants would be both economically viable and environmentally safe.

Crops such as maize have a very high demand for potassium. For example, for a 40 t ha^−1^ fresh yield of maize, ~175.0 kg ha^−1^ potash is required. It has been reported that in China, 20% (2.7 Mt) of the total potash consumption is attributed to maize cultivation (Zhang M. et al., 2023; Zhang S. et al., 2023). In India, 2.61 Mt. of potash fertilizer was consumed in 2019–20, and importantly, the entire requirement of potash fertilizer in the country is primarily met through imports (Indian Minerals Yearbook, 2021). Globally, India ranks among the top five consumers of K fertilizers. As the majority of K fertilizers are imported into India, global price fluctuations and increasing duties lead to higher cultivation costs. Thus, the use of microorganisms to make unavailable K sources available to plants can be a cheaper and ecologically viable alternative to chemical fertilizers. Soil microbes with known roles in the natural K cycle can solubilize insoluble forms of K present in minerals through organic acid production or through chelation of silicate ions, thereby bringing K into the soil solution (Zhang and Kong, 2014; Etesami et al., 2017; Das and Pradhan, 2016; Maurya et al., 2016). Various studies have shown the benefits of K-solubilizing bacteria (KSB), such as *Bacillus mucilaginosus*, *B. edaphicus, Paenibacillus mucilaginosus,* etc. (Maurya et al., 2016; Olaniyan et al., 2022), on the growth and K nutrition of plants. In addition to K solubilization, many such bacteria are endowed with multiple plant growth-promoting traits that may significantly contribute to increasing the total productivity. The rhizosphere and cultivated soils are the most studied ecological niches for isolating such mineral solubilizers. However, forest soils, which represent virgin and unexplored habitats, can also be rich reservoirs of such bacteria due to less anthropogenic disturbance and abundant nutritional resources (Zhang and Kong, 2014; Saha et al., 2016; Meena et al., 2015). Earlier, *Mesorhizobium* sp. was identified as a potential K solubilizer obtained from the temperate and cold desert regions in North India (Baba et al., 2021). *Bacillus, Enterobacter,* and *Pseudomonas* were found to be potential KSB isolated from forest soils (Dong et al., 2019). Identifying microbial strains capable of efficiently mobilizing K from insoluble/recalcitrant sources can help enhance productivity without compromising yield. With these factors in mind, we identified potential KSB from the soils of a tropical evergreen forest in Meghalaya and evaluated their plant growth-promoting abilities under controlled conditions. Due to the substantial potassium (K) requirement and strong yield responsiveness to K availability in maize, it was selected as a sensitive and reliable biological system to assess the efficacy of K-mobilizing microbial inoculants.

## 2 Materials and methods

### 2.1 Isolation of bacteria and screening for potassium (K) solubilization

Soil samples were collected from the Nongkhyllem Wildlife Sanctuary (25.92250–25.92185 °N, 91.86100–91.88096 °E), Meghalaya, which belongs to a tropical evergreen forest, and brought to the laboratory in sterile polythene bags. The collected samples were laterite soil with a pH ranging from 4.5 to 5.5. Plant debris and gravels were removed, and the samples were shade-dried, followed by grinding and sieving to obtain fine powder. This processed sample was serially diluted in sterile physiological saline (0.85% NaCl solution) and plated on tryptose soya agar (TSA) medium (HiMedia, India). The plates were incubated at 30° C for 48 h. All different bacterial morphotypes were sub-cultured on TSA. Purified bacteria were screened for K solubilization by spotting 10 μL of overnight-grown cultures on Aleksandrov agar plates containing potassium aluminosilicate (K_n_Na_12-n_[(AlO_2_)_12_(SiO_2_)_12_]. xH_2_O) and incubating at 30° C for 7 days (Aleksandrov et al., 1967). Isolates showing a clear halo zone around the colony were considered positive for K solubilization and selected for further studies (Supplementary Figure S1). An uninoculated broth served as the negative control, and BioPotash (*Bacillus decolorationis*) (Saxena et al., 2021) was used as the positive control for K solubilization.

### 2.2 Quantification of *in vitro* K solubilization by the bacterial isolates

All K-solubilizing isolates were grown in Aleksandrov broth containing potassium aluminosilicate at 30° C with continuous agitation (120 rpm) for 5 days. The culture suspensions were centrifuged at 6000 rpm for 10 min, and the supernatants were collected. The cell-free supernatant was used to estimate K using a flame photometer (Systronic, Flame Photometer, μC, Type: 128, 4478, India), following the methods described previously by Sugumaran and Janarthanam (2007). A known concentration of potassium chloride (KCl; 20–100 ppm) was used as the standard.

### 2.3 Phenotypic characterization of the bacterial isolates

Bacterial isolates were characterized based on colony morphology using a stereo microscope (Olympus™, SZX47). Isolates were subjected to Gram staining for Gram reaction and cell shape. Physiological characterization of the isolates was done on the basis of growth or no growth on different carbon sources, temperature (4, 15, 35, and 45° C), pH range (5–11), and various salt concentrations (0–12%). Biochemical characterization of isolates was performed as per Bergey’s Manual. Observations were recorded after 24 h of incubation.

### 2.4 Characterization of plant growth-promoting traits

Bacterial cultures were grown in nutrient broth supplemented with L-tryptophan (50 μg mL^−1^) for 48 h at 30° C. The supernatants of the bacterial cultures were collected through centrifugation, and Salkowski reagent [50 mL of 35% perchloric acid (HClO_4_) and 1 mL of 0.5 M iron trichloride (FeCl_3_)] was added to them, as described previously by Bric et al. (1991). Subsequently, the absorbance of the mixture was measured at 530 nm using a spectrophotometer. The IAA concentration in the culture was determined using a standard curve of pure IAA (10–100 μg mL^−1^).

HCN production was estimated as described previously by Lorck (2016). Briefly, bacterial isolates were streaked on nutrient agar containing 4.4 g/L^−1^ glycine, and a Whatman filter paper no. 1 soaked in 2% sodium carbonate in 0.5% picric acid solution was placed on top of the plate. The plates were then incubated at 30° C for 4 days. The appearance of an orange to red color on the filter paper indicated HCN production.

Qualitative screening for siderophore production was carried out on the Chrome Azurol S Agar medium (Sigma) prepared using the method described earlier by Schwyn and Neilands (1987). Chrome Azurol S (CAS) Agar plates were spot inoculated with 10 μL of bacterial suspension and incubated at 30° C for 48–72 h. The colonies developing a yellow–orange halo around them were considered positive for siderophore production.

Quantitative estimation of phosphate (P) solubilization was performed using National Botanical Research Institute Phosphate (NBRIP) broth containing rock phosphate (5 g L^−1^) as a source of insoluble P. Cultures were grown in NBRIP broth for 5 days at 30° C and 150 rpm. After growth, an aliquot of 5 mL was withdrawn and centrifuged at 6,000 rpm for 10 min to obtain the supernatant, which was analyzed for phosphate concentration. Available phosphate was estimated spectrophotometrically in the culture supernatant at 600 nm, following the method described by Fiske and Subbarow (1925). A standard curve using KH_2_PO_4_ (10–100 μg mL^−1^) was prepared, and the amount of P in the medium was expressed as mg L^−1^.

### 2.5 Molecular identification

Overnight-grown bacterial cultures were used to isolate genomic DNA using a NucleoPore Fungal/Bacterial gDNA isolation kit (Genetix, India) following the manufacturer’s protocol. The purity and integrity of DNA were checked through electrophoresis using 0.8% agarose gel. The bacterial isolates were identified by amplifying the 16S rRNA genes using the primer pairs 27F (5′-AGAGTTTGATCMTGGCTCAG-3′) and 1492R (5′-GGTTACCTTGTTACGACTT-3′) (Edwards et al., 1989) following the procedure described by Chakdar and Pabbi (2017). Briefly, 25 μL of the PCR reaction mixture was prepared in a sterile PCR tube containing 12.5 μL of 2X Go Taq Green Master Mix (Promega, United States), μL of 10 μM forward primer (27F), 1 μL of 10 μM reverse primer (1492R), 50 ng μL^−1^ of DNA template, and 8.5 μL of nuclease-free water. The following PCR program was used to amplify the 16S rRNA gene: initial denaturation at 95° C for 5 min, followed by 35 cycles of denaturation at 94° C for 1 min, annealing at 52° C for 45 s, and extension at 72° C for 1 min; and final extension at 72° C for 10 min. Purified amplicons (using THE Wizard SV Gel and PCR Clean-up System, Promega, United States) were sequenced (from SciGenom Lab Pvt. Ltd., Cochin, India) using the Sanger sequencing method (Yang et al., 2016). Pairwise sequence similarity with closely related neighbors was determined using the EzTaxon server^1^ (Chun et al., 2007). The sequences for each isolate were subsequently submitted to GenBank (NCBI) through the NCBI sequence submission portal to obtain the NCBI accession number.

Evolutionary analyses were carried out using the MEGA7 software to construct a phylogenetic tree on the basis of 16S rRNA gene sequences of these four bacterial isolates, along with the 16S rRNA gene sequences of their closest neighbor type strains extracted from Eztaxon (Kumar et al., 2016). The evolutionary history was inferred using the neighbor-joining method (Saitou and Nei, 1987). The confidence level of each branch was tested by bootstrapping with 1,000 replicates (Felsenstein, 1985). Evolutionary distances were computed using the Jukes-Cantor method (Jukes and Cantor, 1969).

### 2.6 Analysis of physicochemical properties of the soil used for the pot experiment

Fine sandy loam soil was used in the pot experiment. The physicochemical properties of the dried and sieved soil were estimated according to standard protocols. The pH of the soil sample was estimated as described by Schofield and Taylor (1955) using a pH meter. Briefly, a soil suspension was prepared by mixing 10 g of soil with 20 mL of deionized water. The mixture was shaken for 20 min at 120 rpm. Subsequently, the suspension was allowed to stand, and the clear supernatant was collected. The pH was estimated by immersing a calibrated pH electrode in the supernatant, and the pH of the soil sample was recorded. The same soil suspension was used to determine the electrical conductivity (EC), as described by Rhoades (1996).

The soil bulk density was estimated according to the protocol described by Resch and Wahbi (2016). Briefly, the soil bulk density was measured by collecting undisturbed soil samples by inserting a metal ring (of known volume) into the soil and determining the weight of the collected soil after drying at 105° C in a hot air oven for 24 h. The bulk density was calculated using the following formula:


$$\begin{array}{l} \text{Bulk density}\;(g\;{\mathrm{cm}}^{-3})=\mathrm{Dry}\;\text{soil weight}\;(g)\\ /\text{Undisturbed soil volume}\;({\mathrm{cm}}^{-3}) \end{array}$$

Further, the soil macronutrients like available nitrogen (N), available phosphorus (P), and available potassium (K) were estimated following the Kjeldahl method, Olsen method, and the methods described by Bray and Kurtz, respectively (Bremner, 1960; Olsen et al., 1954; Bray and Kurtz, 1945).

### 2.7 Pot experiment

Maize (*Zea mays* var. VMH 55) was selected as a test plant to study the effect of KSB inoculation. A pot trial was conducted using fine sandy-loam soil. The soil was sterilized by autoclaving at 121° C for 1 h for three consecutive days. Pre-cleaned pots (1 ft. diameter and 1.5 ft. height) were filled with 3.5 kg sterile soil with moisture content maintained at 60%. For inoculation, maize seeds were surface sterilized with sodium hypochlorite (2%) and rinsed with sterile Milli-Q water. Surface-sterilized maize seeds were soaked in a carboxy-methyl cellulose (CMC, 1%) suspension containing bacterial cells (~10^8^ CFU mL^−1^) for 4 h. The seeds were then dried under airflow in a laminar airflow chamber and sown in the pots. Five seeds per pot and three pots per treatment were maintained. The treatments were as follows: T1 (negative control, no bacterial inoculation), T2 (inoculated with NPH1), T3 (inoculated with NPH2), T4 (inoculated with NPH3), T5 (inoculated with NPH4), and T6 (positive control, BioPotash inoculation). BioPotash is a liquid K-solubilizing liquid biofertilizer developed at ICAR-NBAIM and widely used by local farmers (Saxena et al., 2021). After sowing, pots were kept in a greenhouse under the following conditions: temperature, 25 ± 2 °C; humidity, 60%; light intensity or photoperiod, natural light of a 12:12 h L: D cycle. The plants were harvested after 1 month, and biometric parameters, such as shoot length, root length, shoot fresh weight, and root fresh weight, were recorded. The plant samples were washed with deionized water and 0.1 N HCl, followed by drying in a hot air oven at 60–70° C for 3 days. The dried plant samples were analyzed for P and K uptake.

### 2.8 Plant analysis for phosphorus and potassium

To estimate P and K content, 0.5 g of dried and ground plant samples were first digested with a mixture of HNO_3_: HClO_4_ (10:4). The phosphorus content in the digested samples was determined following the methods described by Jackson (1958). The potassium content in the plant extracts was determined using the flame photometric method (Sugumaran and Janarthanam, 2007).

### 2.9 Hemolytic activity

Hemolysins are one of the important virulence factors that are associated with many of the pathogenic bacteria. The determination of hemolytic activity through the production of hemolysins is helpful in identifying human- or animal-pathogenic bacteria. In the present study, blood agar medium was used to test the hemolytic activity of bacteria. Blood agar base (HiMedia, India) was used to prepare the medium; after autoclaving the medium, 5% sterile sheep blood was added aseptically to the sterile medium cooled to 50° C. The medium was shaken properly to mix the blood homogeneously and poured into sterile Petri plates. A total of 20 μL of a freshly grown bacterial culture was streaked on a blood agar plate and incubated for 3 days at 37° C. The plate was observed periodically to monitor the color change of the colony and clear zone formation around the colony.

### 2.10 Statistical analysis

Replicated (03) experimental data were subjected to analysis of variance (ANOVA) following a completely randomized design (CRD) using SPSS v.16. Duncan’s Multiple Range test was used to measure the significance of means at the 95% confidence limit, and the ranking of treatments was denoted by superscripted letters. The mean values of the treatments with different letters are significantly different.

### 2.11 Accession number/s relate to sequencing data

The NCBI accession numbers of the 16S rRNA gene sequences of the four potential K-solubilizing bacterial isolates used in this study were KX352446, PP968128, PP968129, and PP968130 for *Priestia megaterium* NPH1, *Bacillus tequilensis* NPH2, *Priestia aryabhattai* NPH3, and *P. aryabhattai* NPH4, respectively.

## 3 Results

### 3.1 Isolation, screening, identification, and characterization of K-solubilizing bacteria

**Figure 1:**
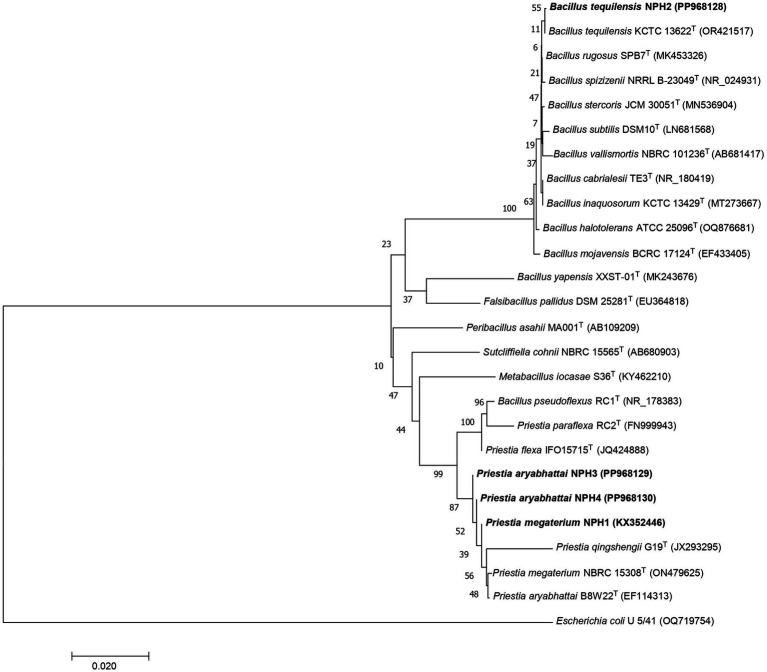
Phylogenetic tree based on the 16S rRNA gene sequences of the bacteria and their nearest neighbors. The tree was constructed by MEGA7 software using the neighbor-joining method with 1,000 replicates for bootstrap analyses.

A total of 61 bacterial morphotypes were isolated from soil samples collected from the Nongkhyllem Wildlife Sanctuary in Meghalaya, India. Screening of these isolates for K solubilization revealed that only four isolates could solubilize K on Aleksandrov agar (Table 1). These bacterial isolates were identified as *Priestia megaterium* NPH1, *Bacillus tequilensis* NPH2, *Priestia aryabhattai* NPH3, and *P. aryabhattai* NPH4 based on 16S rRNA gene sequence identity. Phylogenetic allocation of these four bacterial strains is presented in Figure 1.

**Table 1:** Screening of isolates for K solubilization on Aleksandrov agar.

Sl. no.	Isolate	Clear halo formation on Aleksandrov agar	Sl. no.	Isolate	Clear halo formation on Aleksandrov agar
1	NP1	−	32	NPH2	+
2	NP2	−	33	NPH3	+
3	NP3	−	34	NPH4	+
4	NP4	−	35	NPH5	−
5	NP5	−	36	NPH6	−
6	NP6	−	37	NPH7	−
7	NP7	−	38	NPH8	−
8	NP8	−	39	NPH9	−
9	NP9	−	40	NPH10	−
10	NP10	−	41	NPH11	−
11	NP11	−	42	NPH12	−
12	NP12	−	43	NPH13	−
13	NP13	−	44	NPH14	−
14	NP14	−	45	NPH15	−
15	NP15	−	46	NPH16	−
16	NH1	−	47	NPH17	−
17	NH2	−	48	NPH18	−
18	NH3	−	49	NPH19	−
19	NH4	−	50	NPH20	−
20	NH5	−	51	NPH21	−
21	NH6	−	52	NPH22	−
22	NH7	−	53	NPH23	−
23	NH8	−	54	NPH24	−
24	NH9	−	55	NPH25	−
25	NH10	−	56	NPH26	−
26	NH11	−	57	NPH27	−
27	NH12	−	58	NPH28	−
28	NH13	−	59	NPH29	−
29	NH14	−	60	NPH30	−
30	NH15	−	61	NPH31	−
31	NPH1	+	62	BioPotash	+
			63	Uninoculated broth	−

BioPotash (*Bacillus decolorationis*) was used as a positive control for K solubilization, and uninoculated broth served as a negative control.

All four isolates were able to solubilize K, ranging from 1.62 to 4.71 mg L^−1^ after 5 days of inoculation (Table 2). Bacterial strain *B. tequilensis* NPH2 showed the maximum solubilization of K (4.71 ± 0.17 mg L^−1^) among the bacterial strains (Table 2). All four isolates produced variable concentrations of IAA ranging from 0.23 μg mL^−1^ to 0.46 μg mL^−1^ (Table 2). The maximum (0.46 ± 0.01 μg mL^−1^) IAA production was observed in *P. aryabhattai* NPH3, whereas the least IAA was produced by *P. aryabhattai* NPH4. Although none of the isolates could produce HCN, two isolates, *viz., P. megaterium* NPH1 and *B. tequilensis* NPH2, were able to produce siderophores (Table 2). Quantitative estimation of isolates for solubilization of rock phosphate showed that only *P. aryabhattai* NPH4 was able to solubilize (280 ± 5.2 mg L^−1^) rock phosphate (Table 2).

**Table 2:** Characterization of bacterial isolates for different plant growth-promoting attributes.

Functional properties	NPH1	NPH2	NPH3	NPH4
K solubilization (mg L^−1^)	4.10 ± 0.25	4.71 ± 0.17	1.62 ± 0.12	1.93 ± 0.16
IAA production (μg mL^−1^)	0.42 ± 0.02	0.38 ± 0.01	0.46 ± 0.01	0.23 ± 0.01
Rock phosphate solubilization (mg L^−1^)	No solubilization detected	No solubilization detected	No solubilization detected	280 ± 5.2
Siderophore production	+	+	−	−
Hydrogen cyanide (HCN) production	−	−	−	−

Numerical values presented in the table are mean ± standard error; the “+” symbol indicates that the isolate was positive for that activity. For siderophore activity, “+” indicates orange halo formation in a range of 2.0–3.0 cm; “++” indicates 3.0–4.0 cm; and “+++” > 4.0 cm.

### 3.2 Phenotypic, physiological, and biochemical characterization of KSB

**Figure 2:**
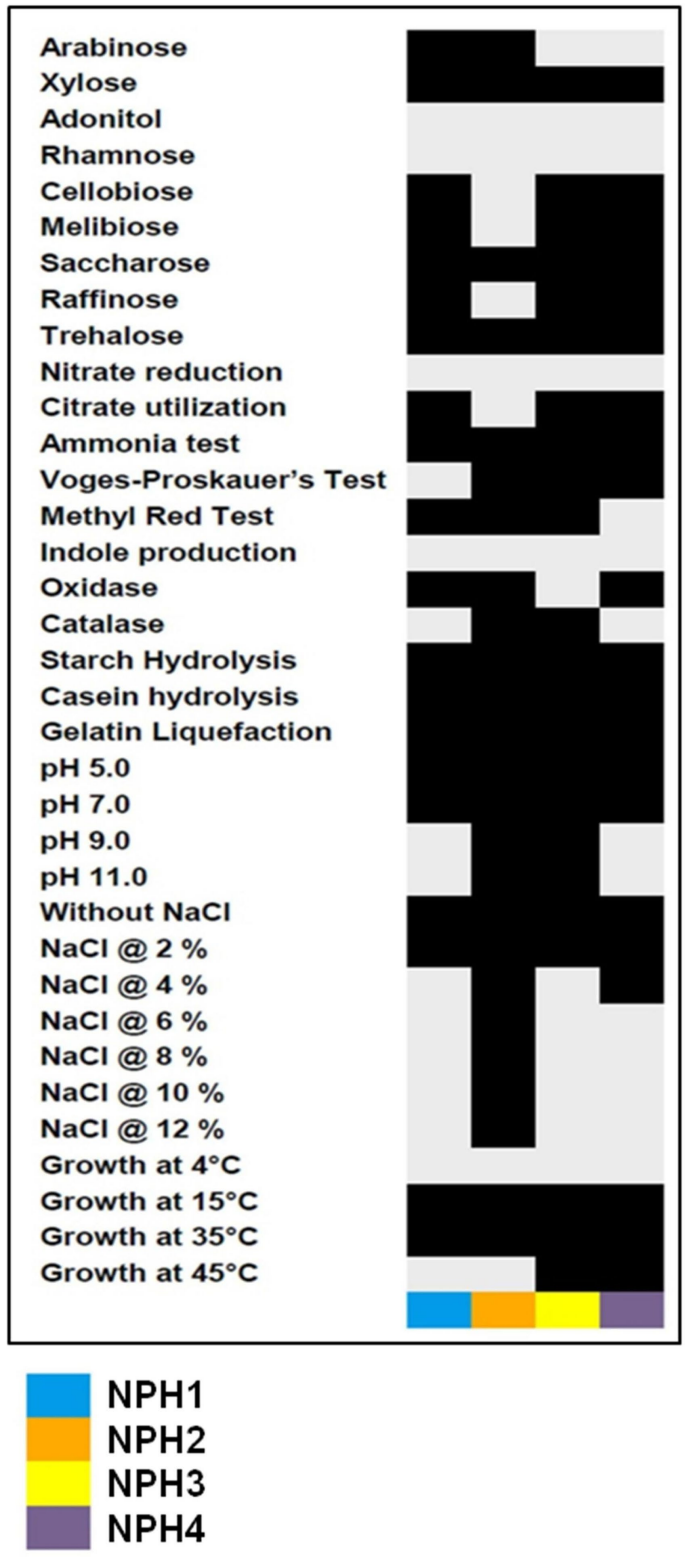
Biochemical and physiological characterizations of potential potassium-solubilizing bacteria. Black boxes represent the presence or positive response to a biochemical and physiological parameter.

Three bacterial strains, *viz., Priestia megaterium* NPH1, *P. aryabhattai* NPH3, and *P. aryabhattai* NPH4 had a waxy texture with yellowish-white pigmentation, whereas *Bacillus tequilensis* NPH2, had a crust-like texture and off-white pigmentation. These four strains were metabolically distinct owing to their varying ability to produce different enzymes and utilize diverse carbon sources (Figure 2). All the strains could tolerate temperatures ranging from 15° C to 35° C; however, two strains, *viz*., NPH3 and NPH4, could grow at up to 45° C. When screened for salinity tolerance, NPH2 could tolerate up to 12% NaCl, 15% KCl, and 12% MgCl_2_. Tests for tolerance to acidity/alkalinity showed that NPH2 and NPH3 could grow in a pH range of 5 to 11, whereas the remaining two could tolerate pH 5 to 7.

### 3.3 Physicochemical properties of the soil used for the pot experiment

The physicochemical properties of the fine sandy loam soil used in the pot experiment were estimated. The soil had the following characteristics: pH, 6.5 ± 0.25; EC, 1.5 ± 0.20 dS m^−1^, and bulk density, 1.485 g cm^−3^. Furthermore, the available macronutrient content in the soil was determined as follows: available nitrogen, 127.0 kg ha^−1^; available P, 26.1 kg ha^−1^; and available K, 137.80 kg ha^−1^.

### 3.4 Effect of bacterial inoculation on growth and nutrient uptake of maize

**Figure 3:**
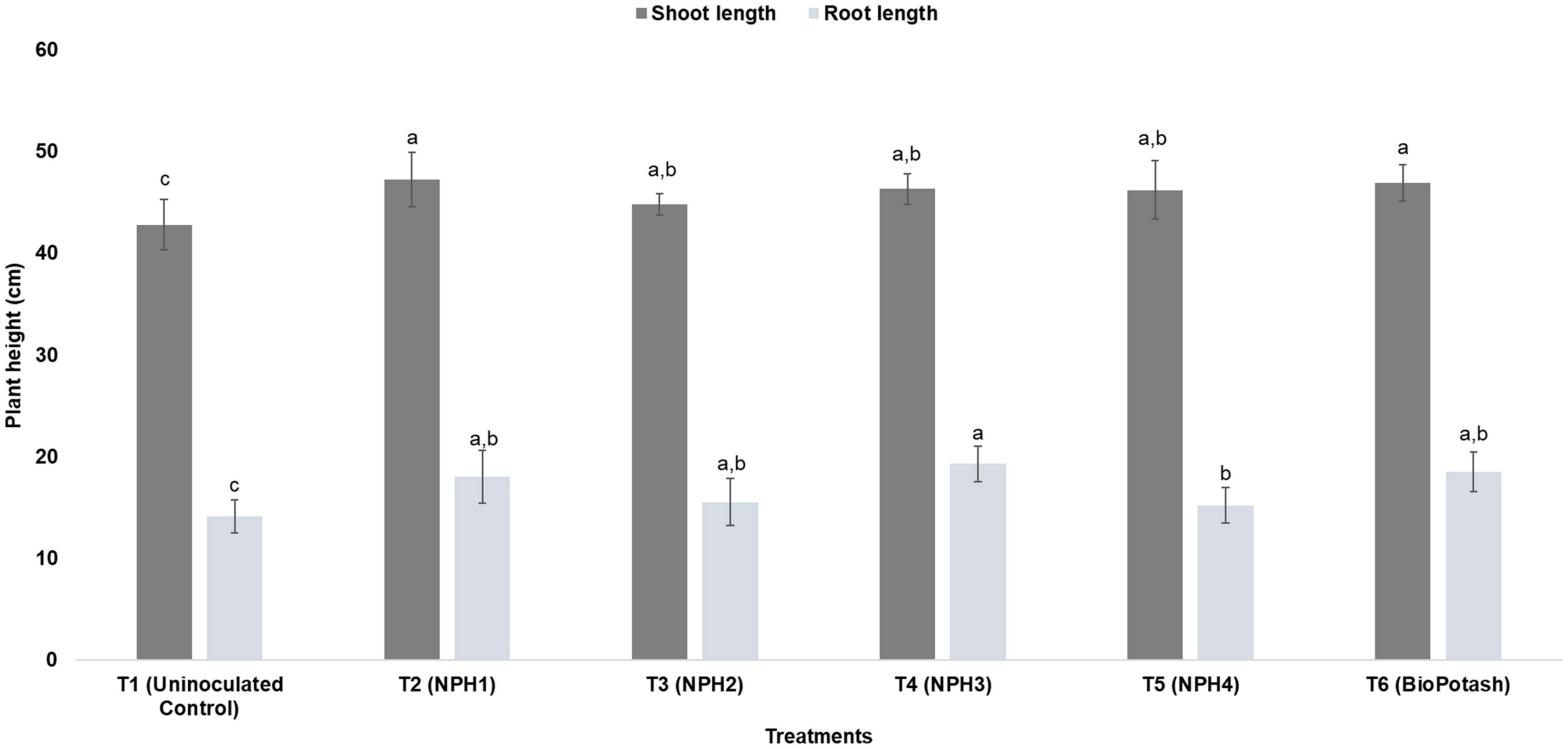
Effect of different treatments on the growth parameters (i.e., root length and shoot length) of the maize plant after 1 month of maize seed treatment. T1: Uninoculated control; T2: Inoculated with NPH1; T3: Inoculated with NPH2; T4: Inoculated with NPH3; T5: Inoculated with NPH4; T6: Inoculated with BioPotash. The columns indicate the mean values, and the error bars indicate the standard errors (SE). The alphabets above each column indicate Duncan’s Multiple Range Test (DMRT) ranks. Means with different letters are significantly different at a *p* value ≤ 0.05.

**Figure 4:**
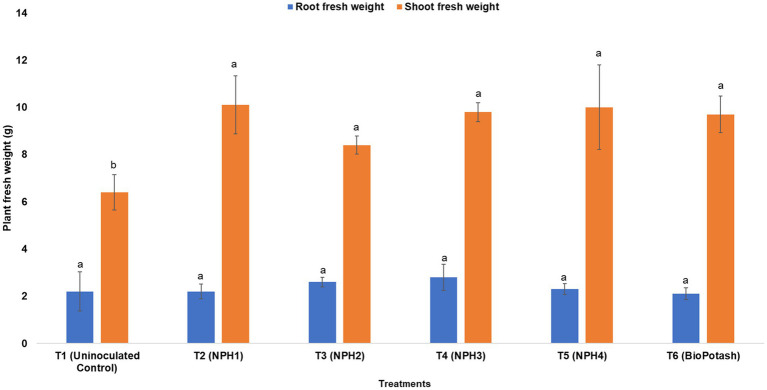
Effect of different treatments on the plant fresh weight (i.e., root fresh weight and shoot fresh weight) of maize plants after 1 month of maize seed treatment. T1: Uninoculated control; T2: Inoculated with NPH1; T3: Inoculated with NPH2; T4: Inoculated with NPH3; T5: Inoculated with NPH4; T6: Inoculated with BioPotash. The columns indicate the mean values, and the error bars indicate the standard errors (SE). The letters above each column indicate DMRT ranks. Means with different letters are significantly different at a *p* value ≤ 0.05.

**Figure 5:**
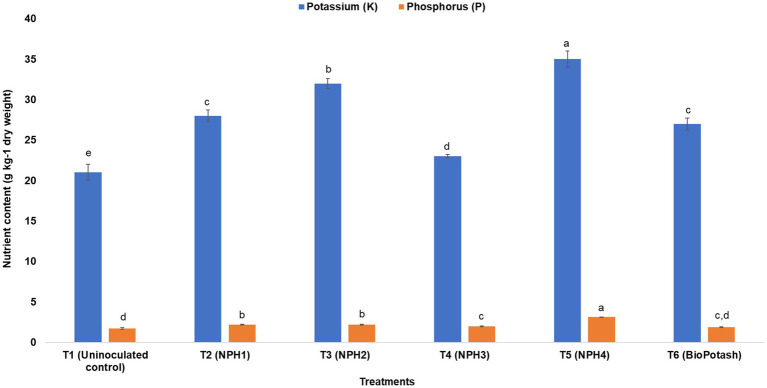
Effect of different treatments on the potassium (K) and phosphorus (P) content in maize plants after 1 month of maize seed treatment. T1: Uninoculated control; T2: Inoculated with NPH1; T3: Inoculated with NPH2; T4: Inoculated with NPH3; T5: Inoculated with NPH4; T6: Inoculated with BioPotash. The columns indicate the mean values, and the error bars indicate the standard errors (SE). The alphabets above each column indicate DMRT ranks. Means with different letters are significantly different at a *p* value ≤ 0.05.

To determine the solubilization potential of K and its uptake by plants, a pot trial was conducted with a maize crop. Treatment of maize seeds with different strains of KSB caused significant changes in plant biometrical attributes. Shoots of maize plants treated with *Priestia megaterium* NPH1 (T2) were significantly longer (47.2 ± 2.68 cm) than those of uninoculated plants (negative control: T1; 42.8 ± 2.48 cm) and were comparable to those treated with BioPotash (positive control: T6; 46.9 ± 1.78 cm). A significant increase in root length was observed for the treatment with *P. aryabhattai* NPH3 (T4; 19.3 ± 1.74 cm) compared to uninoculated plants (Figure 3). Treatments with KSB showed up to a 56% increase in the shoot fresh weight as compared to the negative control (T1); however, no significant difference was observed among the treatments with different bacteria, including the positive control (T6: BioPotash-treated seeds). For root fresh weight, all treatments had similar responses (Figure 4), while all inoculation treatments showed a significant increase in root length compared to the negative control (T1). Analysis of maize plants revealed a 60–80% increase in uptake of P and K (Figure 5) when seeds were treated with microbial inoculants as compared to the negative control (T1). *P. aryabhattai* NPH4-treated plants showed maximum uptake of K (35.0 ± 1.0 g kg^−1^ dry weight) and P (3.13 ± 0.01 g kg^−1^ dry weight) compared to other treatments, including the positive control (Figure 5).

*P. aryabhattai* NPH4, which showed the highest K and P uptake in maize, was screened for hemolytic activity using blood agar medium. No hemolysis was observed, as no clear zone formation was observed around the colony (Supplementary Figure S2).

## 4 Discussion

Microorganisms capable of solubilizing unavailable K-rich minerals, thereby improving the pool of ‘available K,’ hold significant promise as potassic biofertilizers. Considering the high consumption of K fertilizers and the lack of reserves for K-bearing minerals in India and increasing reports of adverse effects of chemical fertilizers on the environment, potassic biofertilizers will play a critical role in the future. The present study aimed to identify potential K-solubilizing bacteria from virgin and unexplored habitats in tropical evergreen forests in Meghalaya, India. Most of the published studies where KSB and PSB have been implicated for plant growth promotion have reported isolation from agricultural soils of neutral to alkaline pH, while only a few were reported from acidic soil (Sharma et al., 2021; Singh et al., 2022; Tripathi et al., 2023; Nguyen et al., 2024). In this work, we identified efficient KSBs with P-solubilizing activities that were capable of improving K and P uptake in maize plants under controlled conditions. All four KSBs identified in this study belonged to two different genera, i.e., *Priestia* and *Bacillus,* and were identified as *P. megaterium, B. tequilensis,* and *P. aryabhattai*. Earlier studies have also reported that the genus *Bacillus* is more potent for K solubilization (Kumar et al., 2012; Gundala et al., 2013). Among the three species, only *P. megaterium* (previously known as *Bacillus megaterium*) has been reported extensively by many workers to solubilize K (Verma et al., 2015; Anjanadevi et al., 2016). *B. tequilensis* has been implicated in P solubilization (Dastager et al., 2011). Seven strains related to *Bacillus megaterium* and *Bacillus coagulans* with both phosphorus and potassium solubilization abilities have been reported from a mountain in Vietnam (Diep and Hieu, 2013). An isolate of *B. aryabhattai* obtained from coastal soils in Incheon, Korea, has also been reported to not only solubilize P and Zn but also to produce IAA and fix nitrogen (Siddikee et al., 2010). The extent of K released by the present isolates was very similar to that reported (Zhang and Kong, 2014), where K solubilization ranged from 0.59 to 4.4 ppm. In another study, *B. mucilaginosus* was recorded with 4.29 mg L^−1^ of K-solubilization in a medium supplemented with muscovite mica, which is similar to the range observed in the present study (Sugumaran and Janarthanam, 2007). Previously, Meena et al. (2015) reported several bacterial K solubilizers showing K solubilization in a range of 2.86 to 16.20 mg mL^−1^.

A diverse group of bacteria with different growth-promoting traits, like production of phytohormones, siderophores, HCN, solubilization of nutrients, and the ability to control the invasion of pathogens, etc., are known to affect plant growth directly or indirectly. However, a single microbial inoculant with multifarious plant growth-promoting traits is considered more beneficial, as a diverse set of attributes can confer an adaptive advantage for survival and colonization of the rhizosphere (Rana et al., 2011). A significant increase in growth of pearl millet was observed by inoculation with *Serratia* and *Pseudomonas* exhibiting multiple growth-promoting traits (Hameeda et al., 2008). Multifarious growth-promoting *Serratia* and *Enterobacter* have been reported to enhance the growth and development of coconut seedlings (George et al., 2013). Improvement of growth of wheat using isolates of *Bacillus* and *Stenotrophomonas* has been reported (Majeed et al., 2015). Growth promotion and disease suppression in ginger were observed by inoculation with multi-trait plant growth-promoting *B. amyloliquefaciens* (Dinesh et al., 2015). In the present study, all the isolates, i.e.*, Priestia megaterium* NPH1, *Bacillus tequilensis* NPH2, *Priestia aryabhattai* NPH3, and *P. aryabhattai* NPH4, exhibited at least two growth-promoting traits, *viz.,* K solubilization and IAA production. Inoculation of *Priestia megaterium* NPH1 and *P. aryabhattai* NPH3 resulted in significant enhancement in root length of maize, which may be attributed to IAA production, as NPH3 and NPH1 were recorded as the highest producers of IAA among the selected strains. *Priestia megaterium* NPH1 and *Bacillus tequilensis* NPH2, on the other hand, showed three PGP traits, *viz.,* K solubilization and siderophore as well as IAA production. Improved root development in the inoculated plants in the present study suggests a possible contribution of the IAA produced by the bacteria. Also, it has been reported that growth and iron uptake in crop plants are improved due to siderophore production by bacteria (Singh et al., 2018; Verma et al., 2022; Zhang M. et al., 2023; Zhang S. et al., 2023). Thus, enhanced shoot and root growth of the maize plants in the present study might be due to a combined effect of hormonal modulation and improved nutrient uptake. *P. aryabhattai* NPH4 showed the maximum number of beneficial traits like P solubilization, K solubilization, and IAA production. Inoculation of the isolate NPH4 resulted in the highest uptake of K (35.0 ± 1.0 g kg^−1^ dry weight) and P (3.13 ± 0.01 g kg^−1^ dry weight) along with improved plant growth compared to the uninoculated control as well as the positive control. Similar results were reported earlier, too (Yu et al., 2012). They reported an increase in K and P uptake when the walnut plant was treated with *Pseudomonas chlororaphis* [28.33 g kg^−1^ (K) and 4.52 g kg^−1^ (P)] and *Bacillus megaterium* [28.07 g kg^−1^ (K) and 4.45 g kg^−1^ (P)]. Rafique et al. (2017) reported enhancement in maize plant growth upon co-inoculation with sawdust biochar and *Lysinibacillus fusiformis* 31MZR, which further resulted in increased nutrient uptake such as N (32.8%), P (72.5%), and K (42.1%). *Pantoea* sp. J-1, *Burkholderia cepacia* Z-7, and *Acinetobacter baumannii* B-6 screened from the maize rhizosphere exhibited varying degrees of IAA production, N-fixation, P-solubilization, and siderophore production and had shown a growth-promoting effect on maize (Luo et al., 2024). It is pertinent to mention here that *P. aryabhattai* NPH4 was the only strain that could solubilize rock phosphate *in vitro*; hence, P solubilization must be the major reason for increased uptake of P. Earlier studies have shown that the strain with IAA production and potash solubilization efficiency results in higher uptake of N, P, and K (Anjanadevi et al., 2016; Sheng and He, 2006). Increased available P and K in the maize rhizosphere due to the presence of solubilizing bacteria, along with improved root growth, might be the major factors for higher uptake of P and K in the plants due to the inoculation of NPH4. Although *P. aryabhattai* NPH4 did not show very high K release from K-Al silicates *in vitro*, it resulted in maximum uptake of K in maize. In light of the available results and published literature, we hypothesize that improved plant K acquisition in maize plants under controlled conditions might be due to the ability of the bacteria to perform better in soil by dissolving a mixture of K-bearing minerals or the induction of the plant transporters for uptake of K. However, this needs further studies to establish the hypothesis. The present study also compared the *P. aryabhattai* NPH4 against BioPotash as a reference biofertilizer under controlled conditions. The BioPotash inoculants are known to augment 10–15 kg K ha^−1^ and increase yield by 1–5% in various crops, including maize (Saxena et al., 2021). In the present study, the K and P uptake in BioPotash-treated plants was comparatively lower than those inoculated with *P. aryabhattai* NPH4. However, the effect on plant biomass (root and shoot weight) was not very different in BioPotash and NPH4 inoculations.

Although field experiments have not been conducted in the present work, the strains identified in this study have the potential to improve maize yield, along with the uptake of P and K. However, future field trials should incorporate direct yield measurements under real agronomic environments and include comparisons with commercially available microbial formulations to strengthen practical recommendations.

## 5 Conclusion

Declining nutrient-use efficiency, degradation of soil health, inefficient water use, climate change, the ever-increasing cost of agro-inputs, and volatile food prices are major challenges for Indian agriculture. Moreover, the indiscriminate use of chemical inputs poses a serious threat to the environment. The use of bioinoculants to augment the availability of nutrients for plants can not only reduce the cost of production by curtailing the use of chemical fertilizers but also reduce their negative influence on the environment and facilitate the sustainable development of Indian agriculture. In the present study, we identified the potential bacterium *P. aryabhattai* NPH4, which can not only promote the growth of maize plants under controlled conditions but also increase the uptake of K and P. Owing to its ability to improve both K and P nutrition in plants, it holds immense potential to be used as a bioinoculant for maize. The results of the present study also indicate that the bacterial isolates can elicit/induce nutrient uptake mechanisms, such as *P. aryabhattai* NPH4, increasing K uptake by maize despite its low K release efficiency from K-bearing silicates *in vitro.* However, this paradoxical observation requires detailed studies on the interaction of the *B. aryabhattai* NPH4 with the maize plant for the uptake of the nutrients and mapping the mechanism of mineral uptake under the application of the bacteria with the maize. Wider application and acceptability of bioinoculants to stakeholders require wide environmental adaptability, which enables them to function efficiently in diverse agro-ecological zones. Considering the moderate environmental adaptability of *P. aryabhattai* NPH4 along with its contribution to the uptake of K and P in maize, it is a suitable candidate for commercial exploitation as a bioinoculant. However, before these strains can be used in farmers’ fields, extensive field validation under different agroecologies is required.

## Data Availability

The datasets presented in this study can be found in online repositories. The names of the repository/repositories and accession number(s) can be found below: https://www.ncbi.nlm.nih.gov/genbank/, KX352446 https://www.ncbi.nlm.nih.gov/genbank/, PP968128 https://www.ncbi.nlm.nih.gov/genbank/, PP968129 https://www.ncbi.nlm.nih.gov/genbank/, PP968130.

## Footnotes

1
http://www.eztaxon.org

## Supplementary material

Supplementary Figure S1 Solubilization of potassium alumino silicate in Aleksandrov agar medium by K-solubilizing bacteria as observed after 4 days of incubation.

Supplementary Figure S2 Pathogenicity test of the isolate on blood agar medium as observed after 3 days of incubation.
