# Supplementary figures and images for: Potassium-solubilizing bacteria from tropical forest soils enhance potassium and phosphorus uptake in maize
Source: Front Microbiol. 2026 May 14;17:1788341. doi: 10.3389/fmicb.2026.1788341 (PMC13220780; doi:10.3389/fmicb.2026.1788341)

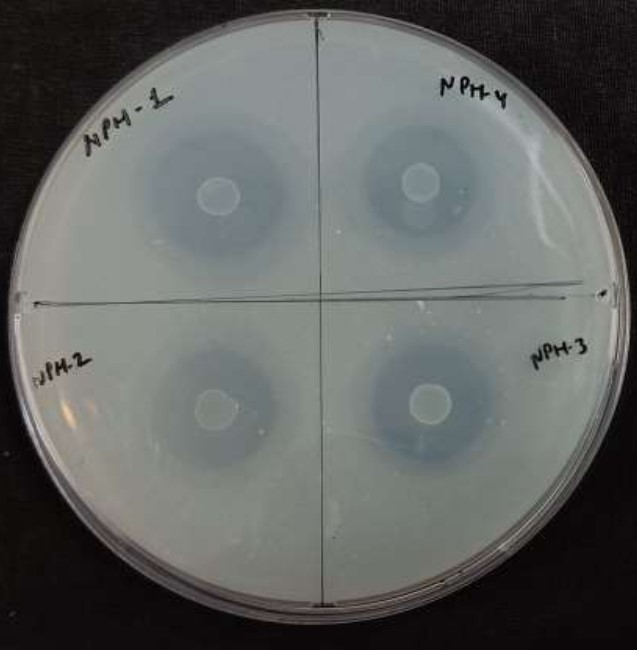

Supplement: Supplementary Figure S1 — Solubilization of potassium alumino silicate in Aleksandrov agar medium by K-solubilizing bacteria as observed after 4 days of incubation. [file Image_1.TIFF]

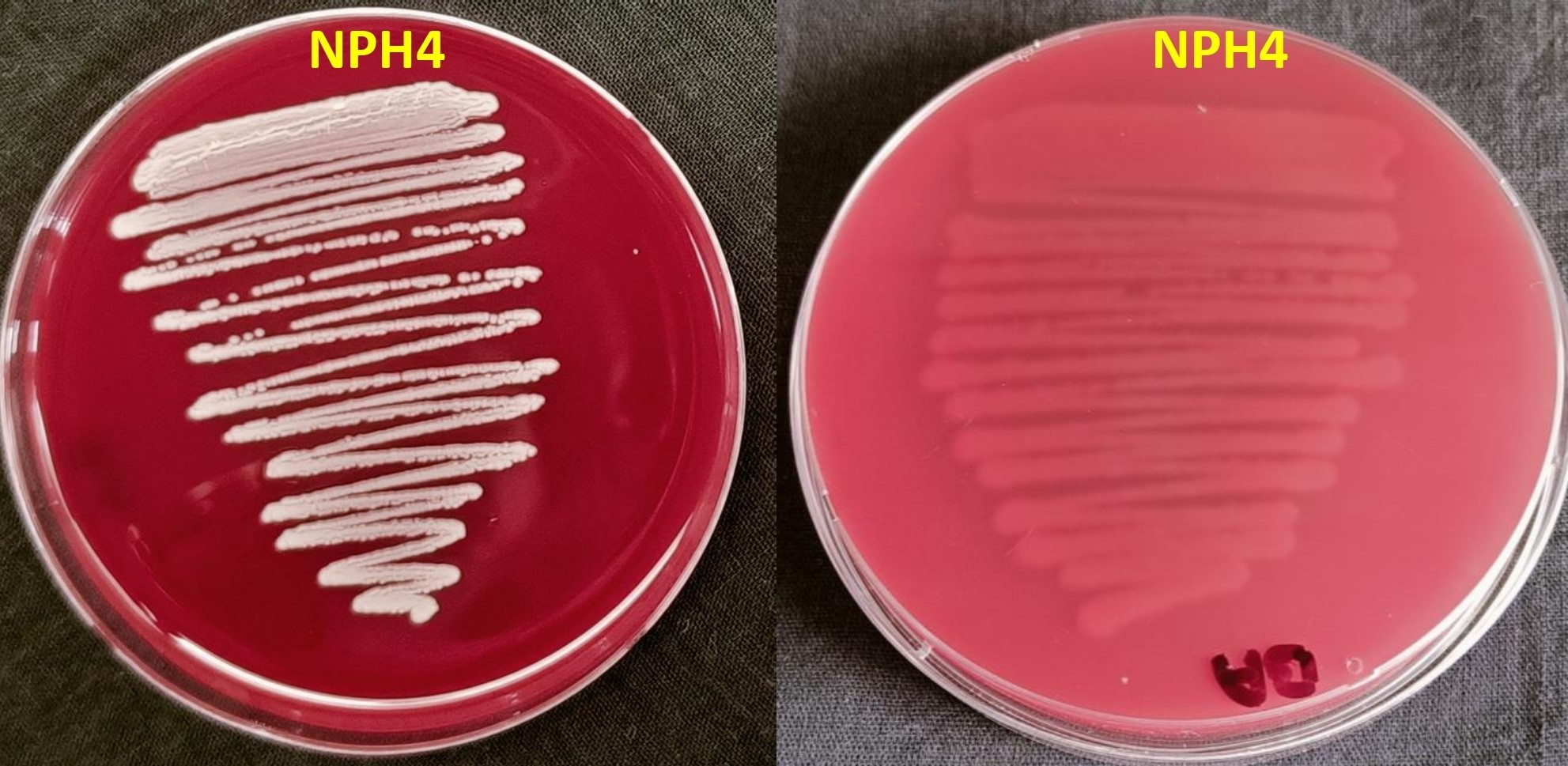

Supplement: Supplementary Figure S2 — Pathogenicity test of the isolate on blood agar medium as observed after 3 days of incubation. [file Image_2.TIFF]
